# Nursing Home Characteristics Associated With Resident COVID-19 Morbidity in Communities With High Infection Rates

**DOI:** 10.1001/jamanetworkopen.2021.1555

**Published:** 2021-03-16

**Authors:** Angela T. Chen, Hyunkyung Yun, Kira L. Ryskina, Hye-Young Jung

**Affiliations:** 1 Perelman School of Medicine, University of Pennsylvania, Philadelphia; 2 Leonard Davis Institute of Health Economics, University of Pennsylvania, Philadelphia; 3 Department of Population Health Sciences, Weill Cornell Medical College, Cornell University, New York, New York; 4 Division of General Internal Medicine, Perelman School of Medicine, Department of Medicine, University of Pennsylvania, Philadelphia

## Abstract

This cross-sectional study uses data from the Centers for Medicare and Medicaid Services Nursing Home COVID-19 Public File to assess the characteristics associated with resident morbidity among 3008 nursing homes in US communities with the highest COVID-19 infection prevalence.

## Introduction

Nursing home (NH) residents have been disproportionately affected by the coronavirus disease 2019 (COVID-19) pandemic. Transmission rates in an NH’s surrounding community have been identified as a key risk factor associated with NH COVID-19 outbreaks.^1^ It is not known whether some NHs within communities are more successful at mitigating outbreaks among residents than others. We examined NHs in communities with the highest COVID-19 prevalence to identify characteristics associated with resident infection rates.

## Methods

This cross-sectional analysis used data on COVID-19 cases in US NHs reported through October 11, 2020, in the Centers for Medicare and Medicaid Services Nursing Home COVID-19 Public File.^2^ The first week of COVID-19 data reporting from the Centers for Medicare and Medicaid ended May 24, 2020. However, NHs could opt to report data retrospectively back to January 1, 2020. We merged these data with the 2017 Long-term Care: Facts on Care in the US (LTCFocus) database^3^ to obtain NH characteristics. We also used the USAFacts website^4^ to obtain county-level infection rates and the 2017 American Community Survey^5^ to obtain community characteristics. The Weill Cornell Medical College Institutional Review Board determined this study to be exempt from review because it did not involve human participants. This study followed the Strengthening the Reporting of Observational Studies in Epidemiology (STROBE) reporting guideline.

Our analysis was restricted to counties in the top quartile of COVID-19 prevalence (mean, 36.0; range, 28.3-164.9 per 1000 population) nationwide. Within these counties, we compared facility characteristics by quartile of COVID-19 prevalence (cases per 1000 NH residents), including resident demographic characteristics (age, sex, and race), and activities of daily living score.^6^ The analysis also includes the number of NH beds, occupancy rate, for-profit status, chain membership, direct care staff hours, presence of an advanced practitioner (nurse practitioner or physician’s assistant), Alzheimer disease specialty unit presence, and the shares of residents covered by Medicare and Medicaid. County characteristics included median household income, percentage of individuals 75 years or older, and rural location. We used 1-way analysis of variance for continuous variables and χ^2^ tests for categorical variables to test for statistical significance (2-sided *P* < .05) of differences in NH characteristics. Multivariable linear regression was used to examine characteristics associated with NH COVID-19 prevalence. We included hospital referral region fixed effects to account for unobserved regional factors that may affect COVID-19 spread. Standard errors were adjusted for clustering at the state level. Stata/IC version 16.0 (StataCorp LLC) was used for analysis.

## Results

Our sample included 3008 NHs (255 923 occupied beds). The full cohort had a mean (SD) age of 78.4 (7.3) years, 165 582 residents (64.7%) were female, 90 341 residents (35.3%) were male, and 158 160 residents (61.8%) were insured by Medicaid. The NHs had a mean (SD) of 6.7 (9.5) COVID-19 cases per 1000 residents in the lowest quartile (755 NHs) and a mean (SD) of 677.1 (146.2) cases per 1000 residents in the highest quartile (752 NHs).

Adjusted estimates indicate that residents in NHs with more COVID-19 cases were older (regression coefficient, 2.2; 95% CI, 0.4-4.0; *P* = .02), the NHs had a lower proportion of White residents (−1.0; 95% CI, −1.7 to −0.2; *P* = .02), and residents had higher activities of daily living scores (7.1; 95% CI, 1.9-12.3; *P* = .009) (Table). In addition, a higher proportion of residents were insured by Medicaid (0.9; 95% CI, 0.1-1.7; *P* = .03), and the NHs had lower occupancy rates (−4.1; 95% CI, −5.1 to −3.0; *P* < .001) and fewer direct care hours per patient per day (−21.9; 95% CI, −32.7 to −11.0; *P* < .001). Nursing homes with more COVID-19 cases were more likely to have an advanced practitioner (33.7; 95% CI, 9.8-57.6; *P* = .007) compared with NHs with fewer COVID-19 cases among residents.

**Table.** Characteristics of 3008 NHs in Communities With the Highest COVID-19 Prevalence^a^

Characteristic	NH COVID-19 prevalence^b^				*P* value^g^	Regression coefficient (95% CI)^h^	*P* value
	Lowest (Q1) (n = 755)^c^	Q2 (n = 750)^d^	Q3 (n = 751)^e^	Highest (Q4) (n = 752)^f^			
NH home resident characteristics, mean (SD)							
Age, y	78.5 (8.6)	79.0 (8.2)	78.0 (6.6)	77.9 (5.4)	.02	2.2 (0.4 to 4.0)	.02
Female, %	65.2 (12.2)	65.3 (12.8)	63.9 (13.3)	64.5 (11.1)	.10	−0.3 (−1.1 to 0.6)	.55
White, %	76.8 (23.8)	71.7 (24.9)	66.5 (25.0)	65.7 (25.5)	<.001	−1.0 (−1.7 to −0.2)	.02
ADL score^i^	16.3 (2.9)	16.9 (2.7)	17.0 (2.5)	16.9 (2.4)	<.001	7.1 (1.9 to 12.3)	.009
Insured by Medicaid, %	59.3 (23.4)	58.4 (25.5)	63.1 (22.8)	66.4 (18.9)	<.001	0.9 (0.1 to 1.7)	.03
Insured by Medicare, %	13.5 (13.2)	15.8 (14.3)	13.7 (12.2)	13.3 (10.2)	<.001	−0.02 (−1.4 to 1.4)	.98
NH facility characteristics							
No. of beds, mean (SD)	99.2 (59.4)	131.9 (79.4)	132.2 (62.8)	122.6 (50.5)	<.001	0.1 (−0.1 to 0.2)	.57
Occupancy rate, mean (SD), %	72.5 (16.3)	72.7 (14.5)	70.9 (14.4)	66.3 (13.5)	<.001	−4.1 (−5.1 to −3.0)	<.001
For-profit, No. (%)	539 (71.4)	533 (71.1)	586 (78.0)	622 (82.7)	<.001	10.1 (−16.9 to 37.0)	.45
Multifacility chain membership, No. (%)	424 (56.2)	404 (53.9)	418 (55.7)	475 (63.2)	.002	4.4 (−15.3 to 24.0)	.65
Direct care, mean (SD), hours per patient-day	3.6 (1.0)	3.7 (1.0)	3.6 (0.8)	3.5 (0.8)	<.001	−21.9 (−32.7 to −11.0)	<.001
Presence of an advanced practitioner, No. (%)^j^	355 (47.0)	399 (53.2)	443 (59.0)	451 (60.0)	<.001	33.7 (9.8 to 57.6)	.007
Presence of Alzheimer disease specialty unit, No. (%)	89 (11.8)	119 (15.9)	116 (15.5)	108 (14.4)	.10	15.9 (−8.8 to 40.5)	.20
Community characteristics							
Median household income, mean (SD), $ (in thousands)	50.2 (11.6)	51.8 (13.8)	50.5 (13.3)	47.4 (11.7)	<.001	0.7 (−0.5 to 1.8)	.25
Population aged ≥75 y, mean (SD), %	6.5 (2.1)	6.3 (1.9)	6.2 (1.8)	6.1 (1.6)	<.001	−3.8 (−15.1 to 7.6)	.50
Rural, No. (%)^k^	185 (24.5)	125 (16.7)	135 (18.0)	177 (23.5)	<.001	6.5 (−32.8 to 45.8)	.74

Abbreviations: ADL, activities of daily living; COVID-19, coronavirus disease 2019; NHs, nursing homes; Q, quartile.

^a^ These communities are the counties in the top quartile of COVID-19 prevalence as measured by cumulative confirmed community COVID-19 cases per 1000 members of the population by county Federal Information Processing Standard Publication code. These cases do not include confirmed COVID-19 cases among NHs in the respective county.

^b^ Lowest (Q1) and highest (Q4) COVID-19 prevalence refer to NHs in the bottom and top quartile of cases per 1000 residents, respectively.

^c^ These 755 NHs had a mean (SD) of 6.7 (9.5) COVID-19 cases per 1000 residents.

^d^ These 750 NHS had a mean (SD) of 84.5 (40.5) COVID-19 cases per 1000 residents.

^e^ These 751 NHs had a mean (SD) of 302.4 (84.2) COVID-19 cases per 1000 residents.

^f^ These 752 NHs had a mean (SD) of 677.1 (146.2) COVID-19 cases per 1000 residents.

^g^ One-way analysis of variance (continuous variables) and χ^2^ tests (categorical variables) were used to assess whether differences across quartiles were statistically significant (2-sided *P* < .05).

^h^ Hospital referral region fixed effects were included in the regression model and the SEs were adjusted for clustering at the level of the state.

^i^ Ranges from 0 (independent resident) to 28 (completely dependent resident).

^j^ The percentage of facilities with a physician extender, meaning a nurse practitioner or a physician assistant.

^k^ The urban/rural classification is defined by the US Census Bureau. An urban region is either (1) an urbanized area of 50 000 or more people or (2) an urban cluster of at least 2500 people and less than 50 000 people. The rural category encompasses all population, housing, and territory not included within an urban area.

## Discussion

In communities with high COVID-19 prevalence, we found significant inequities in infection rates among NHs with larger proportions of racial minority residents and Medicaid participants. In addition, facilities with fewer direct patient care hours were more susceptible to virus spread. Study limitations stem from the use of COVID-19 data reported by facilities and NH characteristics that may not reflect changes in patient demographic characteristics during the pandemic. Nevertheless, these data sets represent the most comprehensive accounting of NH COVID-19 cases and characteristics available as of October 11, 2020. Interventions such as increasing staff support and directing more resources toward NHs with disproportionate shares of racial minorities and Medicaid participants may reduce disparities in COVID-19 morbidity among NH residents.
